# Services on Platform Ecosystems in the Smart Home 2.0 Era: Elements Influencing Consumers’ Value Perception for Smart Home Products

**DOI:** 10.3390/s21217391

**Published:** 2021-11-06

**Authors:** Ruiyang Tang, Yuki Inoue

**Affiliations:** Graduate School of Humanities and Social Sciences, Hiroshima University, Hiroshima 730-0053, Japan; yuinoue@hiroshima-u.ac.jp

**Keywords:** platform ecosystem, smart home, IoT service, modularity, inter-consumer connectivity, value perception

## Abstract

Recently, smart home products have shown signs of rapid development and increasing awareness of smart home platforms. In order to make smart home enterprises enter the era of Smart Home 2.0, it is necessary to consider the elements related to smart home platforms. This study examines the relationship between consumers’ value perception and the platform ecosystem theory and how this relationship contributes to their perception of smart home products’ value. This study aims to reveal the influence of smart home platform elements on the value perception of consumers regarding consumers’ perception of the smart home products’ value. To achieve this goal, an online survey (n = 595) was implemented to collect data from Japanese respondents. The analytical results presented in this study indicated that consumers, who sense the value of modularization of smart home products and inter-consumer connectivity, can sense the value of smart home products. In addition, consumers who can perceive the value of a platform service can indirectly feel the value of smart home products through modularity and inter-consumer connectivity. The results presented in this study provide new insights into product development in Smart Home 2.0.

## 1. Introduction

### 1.1. Research Background

The rapid development of the Internet of Things (IoT) has led to the increase in smart home-related technologies in recent years due to the later having the most contact with general consumers among various IoT products [1]. Consequently, it has also led to the increase in the number of smart home platforms. For example, Amazon’s Alexa, in the U.S., uses Alexa smart products as its core to provide a platform which enables the connection of smart home products [2]. Another example is Panasonic which provides a platform to control its air conditioners and relies on the platform to offer various types of products. At present, there are other companies developing smart home platforms at the system level. For instance, Google created Fuchsia OS and Huawei created Harmony OS [3].

Through the development of technology which enables one to reach a larger variety of consumers, smart homes have gradually upgraded from Smart Home 1.0 to Smart Home 2.0. In the Smart Home 1.0 era, smart home products received and executed signals through various communication technologies. The related communication equipment in the room can execute the corresponding function by receiving instructions. This effectively serves users and reduces their labor force [4,5]. In contrast, the Smart Home 2.0 era allows the effective combination of technologies such as computers, network communication, and electrical appliance control. It also integrates technologies for the ease of home life, such as home intelligent control, information exchange, and consumer services. This way, the Smart Home 2.0 era helps maintain harmony and coordination between these home facilities and the residential environment. In parallel, the Smart Home 2.0 enables realizing efficient, comfortable, safe, convenient, and personalized family life [4,5,6]. Smart home products, in the Smart Home 1.0 era, are needed solely to realize their individual characteristics. In contrast, the Smart Home 2.0 era emphasizes the need for a smart home platform ecosystem. For example, this includes the management of smart home products and communication between products and consumers. The platform ecosystem perspective is an essential tool in order to achieve this goal [7]. This perspective enables the usage of companies as a group based on smart homes as an ecosystem instead of considering a product-based perspective or enterprise-by-company perspective. Research into smart homes from the perspective of the platform ecosystem can better assist in analyzing and understanding the development direction of smart homes.

The elements of the platform ecosystem and their usage are not widely known due to previous researchers mainly focusing on consumer demand for smart home platform products [8,9] and the smart home as a platform. Gunawan et al. (2018) proposed that through the smart home platform, consumers can exchange data between applications through network services, and users can view related data while controlling household appliances [10]. Kuwari et al. (2018) proposed that the integrated design of a sensor and monitoring system can be used to improve consumer experience in smart home platforms [11]. However, based on the characteristics of the Smart Home 2.0 platform, the connection between products and between products and consumers should be strengthened. Thus, it is necessary to further study the smart home platform ecosystem, instead of only focusing on the smart home platform. 

The platform ecosystem is an “economic community” supported by the foundations of interactive organizations comprised of three elements: platform owners, complements, and consumers [12]. When new technologies appear on the market, some companies will choose to create or join a platform ecosystem to enhance their competitiveness [13]. In the smart home market, the platform owner develops a platform for other members (complements) in order to provide them with products on the smart home platform [3]—for example, HomeKit of Apple, HiLink of Huawei, etc. Complements are companies that provide various products adapted to the platform [7], such as Philips Smart Electronics or Toshiba Home Appliances. Consumers are the end-users who buy and use smart home products. 

### 1.2. Research Question and Purpose

Research on an ecosystem for smart home product platforms is still underway, replete with multiple research perspectives. The motivation of this study is to figure out whether elements of platform ecosystems could be effective in smart home product markets. More specifically, the focus of this study is to determine whether or not consumers perceive value in the potential benefits of realizing a platform ecosystem in smart homes. In other words, this study proposes the following research question. 

RQ: Do consumers, who sense the value provided by each element of the platform ecosystem, perceive the value in smart home products?

Solving this problem is fundamental in order to understand whether the implementation of Smart Home 2.0 can help expand the smart home market. Therefore, the purpose of this study is to reveal how consumer perceptions of the value of various elements related to the platform ecosystem can impact their perception of the value of smart home products.

### 1.3. Novelty and Contribution of This Study

In previous studies, research on the smart home mainly focused on the impact of smart home products on consumers, or contributed to the development of smart home products by studying consumers’ needs [14,15,16]. There have been studies which focused on certain components of the smart home [16,17]. Studies on the smart home platform have mainly focused on the impact of platform owners and complements such as the development and com-position of the smart home platform and the impact of the smart home platform on consumers, etc. [4,18]. Smart Home 2.0 strengthens the connection among products and consumers on the basis of Smart Home 1.0, so it is necessary to introduce the concept of a platform ecosystem for future studies [5,6]. However, there is no comprehensive research from the perspective of platform ecosystem elements. Taking consumers as the research object, the research methods are to collect data through questionnaires’ survey for factor analysis and using the SEM to analyze the data [15,19]. 

This study distributed an online questionnaire, and 595 Japanese consumers in the smart home products market were chosen as the sample target of this study. Thereafter, three elements of the platform ecosystem (platform service, modularity, and inter-consumer connectivity) and consumers’ value perception on smart home products were investigated through this sample. The constituent conditions of the model smart home platform eco-system also meet the needs brought by Smart Home 2.0. On this basis, combined with previous studies, and based on the questionnaire survey data, factor analysis and SEM were used to analyze the results. The results showed that consumers who sense the value of modularity of smart home products and inter-consumer connectivity can sense the value of smart home products. The sense of value of the platform service was not directly related to the sense of value of smart homes. However, platform service showed indirect impacts on consumers’ value perception of smart home products through modularity and inter-consumer connectivity. This study provided further understanding of the relationship between the elements of the platform ecosystem and consumers’ perception of value for smart home products. 

The novelty of this paper lies in the exploration of the smart home from the perspective of the platform ecosystem and using the elements of the platform ecosystem to study consumers’ perception of value in contrast to previous studies which focused on the impact of the smart home on consumers and the development of the smart home platform. Research on the impact of the smart home platform ecosystem on consumers’ perception of value is little. The lack of understanding the smart home platform ecosystem will hinder the smooth development of Smart Home 2.0. Therefore, in this study, the smart home was analyzed from the perspective of platform ecosystem elements, with the intention of further developing literature on smart home research. 

## 2. Literature Review

This section focuses on previous studies related to smart home products and platforms. Following it is an explanation of the concept of the platform ecosystem and its constituents.

### 2.1. Smart Home

#### 2.1.1. Smart Home Products

Smart home refers to the application of information and communication technology (ICT) in home control from the control of home appliances to the automation of home functions (windows, lighting, etc.) [18]. Nowadays, smart homes are part of IoT, which refers to the “things” around us that function through ICTs, sensors, and networks [20]. The Internet of Things technology can bring new value propositions, value transfer and value capture to the smart home industry [21,22]. Bundling different “things” and systems supports the integration of knowledge, technologies, and communication solutions in different fields [20]. Smart home products include Alexa, Google Home, and Sharp’s COCORO series products. At present, smart homes provide an even wider range of services in people’s lives, including auxiliary living, health checks, and safety [14].

In recent years, smart homes have not only focused on energy saving but also on the compatibility and development of intelligent systems [15,19,23]. Smart home technology makes it easier for people to support users to detect and control home devices, thereby improving the quality of life [24]. For example, Wang et al. (2010) suggested that the ZigBee protocol converter was used in the control center to universally control smart home systems [25]. Subsequently, the development of IoT propelled the development of smart homes. Jie and Pei (2013) proposed a solution for integrating many applications into the system through a unified interface [26]. Kadam et al. (2015) claimed that consumers are able to use smart devices to ensure security, energy savings, and ventilation, as well as build smart kitchens and other features of smart homes [27]. The spread of the coronavirus disease worldwide during 2020 also had an impact on smart home sales. Nevertheless, according to projections by the U.S. statistics agency IDC, sales are expected to increase by over 4.1 percent [28]. Based on the prediction of Grand View Research, an American market research institute, the global smart home market will reach USD 476.1 billion by 2020 [29].

In parallel, the difference between “service” and “product” is becoming smaller over time for the benefits of consumers [30]. Modern products, including service functions, have become service products. A service product represents a user’s demand to request services. Service quality helps improve user value, thereby improving user satisfaction, purchase intention, and brand loyalty [31]. Currently, service products are based on the requirements of Smart Home 2.0. According to these requirements, multi-industry and multi-field joint cooperation will promote harmony and coordination between smart home products and the living environment, as well as improve the type and quality of smart home services [4].

#### 2.1.2. Smart Home Platform

In the IoT era, value creation evolved from a single company to a multi-company platform [32]. In the near future, many people will live in a “smart home” which will be an intelligent and coordinated platform ecosystem consisting of software and hardware [33]. As for smart home platforms, communication equipment and technology are used to connect with other devices at home to realize various functions [16]. In IoT platforms (such as smart homes), the network effect resulting from technology configuration between products further increases the value of the platform and allows more companies to participate in it [34]. In the case of a smart home platform, products with platform functions can be provided so as to centrally manage smart home products, including other companies in the platform. 

With the increase in available services provided by the complements, the extensibility of functions has become an important trend in the IoT system. Platform companies may also provide support and consultation for other companies’ products to promote the integrated use of their products and smart home products. With the means of effective platform control, complements can actively contribute to the platform and actively assist other complements in the platform to meet the needs of more consumers [35]. Due to the data being retrieved from various devices, the platform can also be used by complementary companies to collect it, so as to innovate products between companies and even outside the industry. The platform will gradually integrate these technologies or functions so that the complementary ones can work together.

### 2.2. Platform Ecosystem

The platform ecosystem evolved from the concept of the business ecosystem proposed by Moore (1993) [36]. Gawer (2014) emphasizes that various species, such as platform owners, complements, consumers and market intermediaries, constitute the communities of economic destiny [7]. The platform ecosystem is a community coordinated by platform owners, external complements, and consumers [37,38,39]. Suppliers that provide various types of products compatible with platform technology on the platform are called “complementors” [40]. Gawer (2014) believes that the platform ecosystem is an integrated system of complementary modules between platforms [7]. The indirect network effect is an important mechanism of the platform ecosystem [41]. This effect usually occurs in two-sided markets [38]. In other words, as one party’s scale increases, the other party’s interests increase as well [42]. In the platform ecosystem, complementary units use platform technology to develop and deliver complementary products. Consumers can then buy these complementary products through the platform. The platform ecosystem encourages consumers with different needs to adopt the platform they use [43].

This study will introduce three factors related to the platform ecosystem which can generate benefits for the smart home market. 

#### 2.2.1. Platform Service

The platform itself can provide consumers with a large number of services and products. Platform services are the focus of consumer demand [44]. However, platform service has an important influence on cross-platform service quality, and services can be adjusted within a specific platform [45]. One of the platform services is to collect user data in order to establish a valuable connection between the platform and the consumer, thereby further meeting the needs of a consumer [46]. In other words, platform service can generate value for consumers. Through platform service, consumers can create value for themselves and other users through interaction with the platform they use [30]. This not only provides users with higher-value products and services, but also improves users’ awareness of product value [30]. The services of the platform play a leading role in meeting the consumer’s needs [47]. Therefore, platform service could improve the service quality for consumers and help them perceive more value in products and services. 

#### 2.2.2. Modularity of Products

As mentioned in Section 2.2, suppliers who provide various products to the platform are called “complements” [48]. These complements can be modularized to provide consumers with products that can complement each other [30]. Modularization is the expertise of the complementors to produce various products which boost the differentiation and complementarity of products [49]. Thus, allowing consumers to select various products more personally [50]. The network effect between consumers and complements is known as an “indirect network effect” [7] being mainly generated due to complementarity between products. The more complementary the products of a platform, the greater the demand for this specific platform in the market is [7]. 

The main goal of modularity is to increase customer value by creating a more competitive product portfolio [51]. Modular products can improve the scalability of a single product, and the number of complementary products will increase accordingly in a way that products can better serve consumers and meet their needs. Modularization allows developers to improve the functionality, extensibility, and service quality of products for consumers. It can also promote cooperation among complementary partners through this platform [49]. Modularity enables consumers to enjoy better quality and more efficient products and services. In other words, modularized smart home products may be able to better meet the needs of these consumers. Modularization could improve the specialization and configuration of smart homes and improve the consumption experience. 

#### 2.2.3. Inter-Consumer Connectivity

Consumers are the users of products (end users) and one of the components of the platform [52]. The platform is a special market through which consumers can exchange activities with each other [7]. Lee and Robbins (1998) define social connections as a sense of belonging or intimacy in interpersonal relationships [53]. When many consumers use platform products, the characteristics of promoting cooperation among consumers have a direct network effect if the platform has a function to drive it [7]. By then, users can contribute by participating in value creation [54]. The direct network effect refers to the increased value of the service with an increase in the number of users [55]. In other words, when users create value for other users, the network effect is produced [56]. Therefore, it is considered that the cooperation between users’ smart home devices could provide social value for them, as well as increase their sense of use of these products during this process of cooperation.

## 3. Methodology

Online questionnaire surveys were administered to consumers. The data gathered was later grouped and analyzed using a quantitative analysis approach. For this purpose, the consumers’ degree of sense on the value of smart home products (hereafter, it is referred as value perception) was set as the dependent variable. The model design, presented in this study, was based on the definition of the platform ecosystem [7,37,40] and the viewpoint of the smart home platform [1,4,10]. The three elements of the platform ecosystem—“platform service”; “smart home products modularity”; and “inter-consumer connectivity”—that may be related to smart home products were set as explanatory variables. After analyzing the four variables using factor analysis, structural equation modeling (SEM) was employed.

### 3.1. Analytical Model

This study considered three elements of a platform as functionalities in the analytical model in addition to the dependent variable:

- Value perception: Customers’ value perception is generated from their experience of a complete product or service [57]. In other words, consumers perceive the value of their products and services through the abilities of the same products and services to meet their needs. Therefore, value perception is defined as whether products meet the needs of consumers and whether consumers perceive the value of these products.
- Platform services: Platform services are now at the center of meeting consumers’ needs [44]. Platform services support other elements of the platform and help provide better products and services for consumers. Consumers can satisfy their needs and perceive the value of products by using platform services [46,47]. Platform service is a service that can provide platform capabilities for products—all while guaranteeing interconnection and operation among smart home products. Platform services may also provide support and consultation for other companies’ products to promote the simultaneous use of their products and smart home products.
- Modularity of products: The products made by complements and consumers are related by indirect network effects, making consumers feel valued [7]. Complements can also improve the performance and service of products developed through modularization, thus becoming of great value to consumers interested in modularization [49]. Therefore, this study examined the modularization of products. Here, modularization is defined as a combination of smart home products with individual, separated product features. The unification of smart home products refers to a form in which a unified smart home service can be received by a single product.
- Inter-consumer connectivity: Consumer’s interaction can be achieved using a platform [7]. Consumers can meet their own needs by communicating with each other, thereby improving their satisfaction [58]. This way, the smart home platform will play the role of an ecological product combination and meet the needs of consumers. In parallel, consumers can choose products that they think are valuable in order to meet their needs, thus making the interaction between consumers with the same demands and needs more valuable. Hence, this study includes inter-consumer connectivity as a variable. Here, inter-consumer connectivity refers to the ways in which consumers can exchange opinions and communicate with each other through a smart home platform.

**Figure 1:**
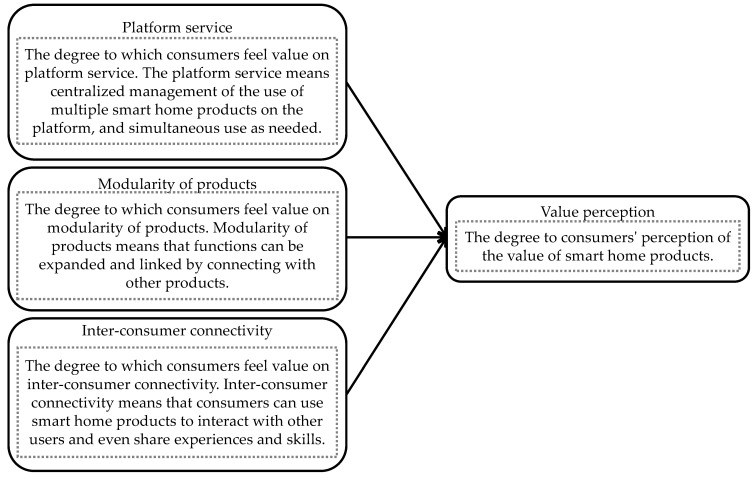
Analytical model.

In summary, the platform ecosystem consists of interrelated service sets through which users can meet various needs in a comprehensive experience [59]. The platform owner is the source of the platform service in smart home platforms [44]. Modularization is the performance of the complements in the platform ecosystem [7] and inter-consumer connectivity is realized through the interactions among consumers [55]. Figure 1 depicts the analytical model used to examine whether consumers who can perceive the value of the three elements of the smart home platform ecosystem can also perceive the value of smart home products or not.

### 3.2. Survey Overview

This study used an online questionnaire survey. The survey took place on 9 February 2021 until 16 February 2021, as within 1 week, enough samples for data analysis were gathered. Macromill, a Japanese questionnaire survey company, was used in order to distribute the questionnaires online. Sampling was randomly made by a survey company in the Japanese market to ensure the randomness of the samples in use. The age group of the samples was between 20 and 65 years old. The minimum samples data to ensure reliability of the results was calculated using the Cochran’s sample size formula [60,61]. The calculation was made as follows. According to a survey conducted by the Statistics Bureau of the Ministry of Internal Affairs and Communications of Japan in April this year, the population aged between 20 and 65 was approximately 68.597 million in 2021 [62]. Thereafter, the reliability was set to 95%, the allowable error was set to 5%, and the response rate was set to 50%. The calculation of all these factors showed that the sample size needed is 385 people. In order to ensure sufficient samples, the expected sample size was set as 800, approximately the double of 385. Assuming that consumers who use smart homes account for 10% of the total sample, it was considered that the desirable number of survey samples should be over 8000 before initial screening stage. Ultimately, 11,029 respondents participated in the questionnaire survey. Then, the details of the survey contents and screening procedure of samples was explained to these participants. 

**Figure 2:**
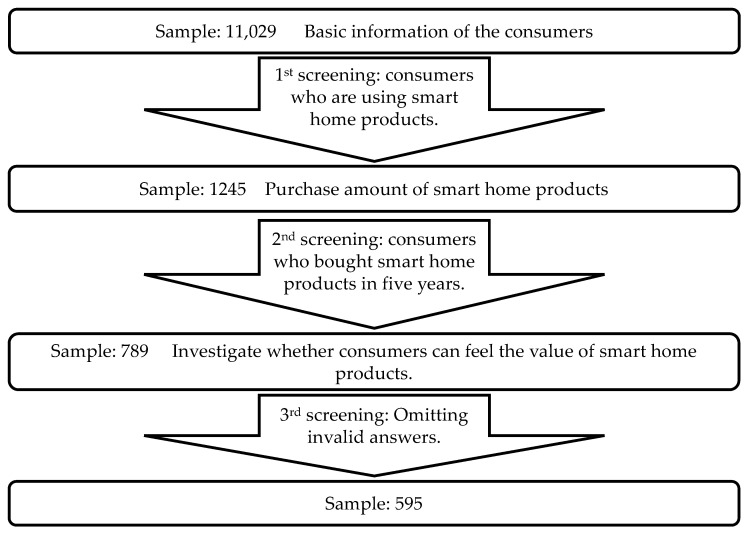
Analytical model of sample screening.

Figure 2 “Analytical model of samples screening” shows the method used for screening the samples gathered for this study. The questionnaire consists of four parts and only those who pass the first screening can continue to the second part. Those who pass the screening of the second part can continue to the third part. The participants who pass the screening of the third part can continue to the fourth and last part of the survey. The survey was distributed to Japanese consumers (at this stage, n = 11,029) in order to examine whether consumers can feel the value of the ecosystem elements of smart home platform. The first part of the survey examines the basic information of the consumers who took part in this specific survey. For example, this included gender, age, birth, occupation, annual income of family, etc. Then, the participants of the questionnaire were screened by the question “Are you using smart home products at present?”. Consumers who answered “I am using it” continued to the second part of the questionnaire survey. Based on the screening, 1245 consumers used smart home products, accounting for 11.3% of the total number of the participants. Later, the participants were screened in the second part by their answer choices to the question of “How much did you spend on smart homes products within the last five years?”. Among the participants in this part, only 789 consumers bought smart home products within the last five years. The third part of the survey examined how these 789 consumers used and bought smart home products, and whether they could feel the value of smart home products. Here, 194 participants were excluded due to invalid answers. The remaining 595 consumers became the object of examination in this study. Even if this sample size was smaller than the initial expectation (=800), it exceeds the required number of samples (>385), ensuring the authenticity of the analysis. Finally, three aspects were selected through relevant elements of the smart home platform ecosystem, namely “platform service”; “modularity of products”; and “inter-consumer connectivity”. Each aspect was tested with specific examples and included 6 questions per aspect. The design of the questionnaire is provided in Section 3.3. 

The characteristics of the 595 respondents were as follows: the sample comprised 407 men and 188 women, accounting for 68.4% and 31.6%, respectively. The age range was 22–65 years. The consumers were divided into three levels based on their annual household income: an annual income of less than 6 million was reported for 225 respondents (37.8% of the total participating consumers); between 6 million and 12 million, for 248 respondents (41.6%); and over 12 million, for 60 respondents (10.1%). Sixty-two people did not fill in the annual income, accounting for 10.5% of the total data. Follow-up data were excluded from the survey. Among the participating consumers, 217 were single (36.4%) and 378 (63.6%) married.

### 3.3. Questionnaire Design

The questions of the survey were designed in a five-point evaluation of one-to-one comparison. In order to ensure the feasibility of the content, the survey was originally written in Japanese language under the supervision of a Japanese native speaker. The development of scales followed the corresponding literature. The items of the questionnaire listed in the Appendix A were translated into the English language. 

“Value perception” consists of six items which draw lessons from previous surveys on consumer demand and studies related to consumer value perception [18,19,24]. We refer to the interpretation of value perception given in the previous research, such as the preference and acceptance of smart homes, to design the items in combination with real-life usage scenarios. This covers the kinds of scenes in which consumers use smart home products. For details regarding the items on value perception, please refer to V1–V6 of Table A1 attached in Appendix A.

“Platform service” consists of six items which draw lessons from previous research on the development and composition of the smart home platform [63,64,65]. The smart home platform can control smart home products or provide services [64]. The smart home platform center processes the data of the smart home through Cloud services and provides corresponding support [65]. Therefore, in this research, we focused on “services established by centralized management of platform” as a platform service. We designed these items by combining the characteristics of platform service development in the previous research with the actual usage scenarios. The design of the items was carried out through consumers’ value perception of services achieved by the centralized management of the platform. For details concerning the items on the platform service, please refer to P1–P6 in Table A1 attached in Appendix A.

“Modularization of Smart Home Products” consists of six items which draw lessons from previous research on modular product development of the smart home [18,66]. Modularization can expand functions through wireless connection [66]. Modularity can complete more complex functions by adding products [18]. We designed these items by combining the characteristics of modular products’ development in the previous research with actual use scenarios. The design of the items was carried out through consumers’ perception of definition of modular products and the methods of functions’ expansion. For details about the items on modularization, please refer to M1–M5 in Table A1 attached in the Appendix A.

“Inter-consumer connectivity” consists of six items, which draw lessons from the previous research on smart home product development and consumer demand for smart home [65,67]. Consumers can share information through products [65]. In regard to privacy-related information, it is easier to share and communicate with family and friends, and for strangers, it is some non-private information [67]. We refer to the interpretation of interaction between consumers given in the previous research, such as the preference and acceptance of smart homes, to design the items in combination with real-life usage scenarios. We tested consumers’ perception of inter-consumer connectivity from the functions that can be used together and can generate interaction between consumers. The design of the items was carried out through consumers’ perception of the functions that can be used together and can generate interaction between consumers. For details related to the items on inter-consumer connectivity, please refer to I1–I6 of Table A1 attached in Appendix A.

### 3.4. Dependent Variable

Based on Figure 1 in Section 3, the dependent variable is “value perception” which is associated with consumers’ needs. In other words, it is concerned with whether the products meet the needs of the consumers and whether these consumers can perceive the value of these products. In this study, six items were used to meet the needs of consumers, and a one-to-one five-point evaluation was conducted. Factor analysis was used to confirm the consistency of the questions, and subsequently, the factor “value” was created. 

### 3.5. Explanatory Variables

A five-point evaluation of one-to-one comparison was conducted based on the three variables presented in this study—platform services, modularization of smart home products, and inter-consumer connectivity. Each of these variables was put to test using factor analysis in order to confirm the consistency of questions, and subsequently create each of these factors. The definitions of these variables are presented here below.

- Platform service: This variable represents the acceptance of platform services by consumers, that is, the element of the “platform company”. This variable is referred to as factor “Platform”;
- Modularization of smart home products: This variable refers to the degree of consumers’ preference for the modularization of smart homes. This variable is referred to as factor “Modularization”;
- Inter-consumer connectivity: Interactions between users among themselves concerning products are realized and enable consumers to perceive the value of these products. This variable represents consumers’ interactions. This variable is referred to as factor “Interactive”.

## 4. Results

### 4.1. Results of the Factor Analysis

Results concerning the analysis of consumers’ value perception are presented in Table 1 here below. The results of Table 1 show that for the objective variable, the factor loading of “Value” ranged from 0.60 to 0.72 out of six items. For the explanatory variables, the factor loading of the six items of the “Platform” ranged from 0.63 to 0.80. One question item in “Modularity” was excluded due to the lack of consistency and the factor loading of five items in “Modularity” ranging between 0.47 and 0.77. The loading of the six items of “Interaction” ranged between 0.68 and 0.73. In this factor analysis study, we refer to the previous studies on consumers’ perception of the value of smart home, such as whether the characteristics of the smart home meet the needs of consumers, and use factor analysis to verify the reliability of the data [19]. Therefore, we used the following three reliability indicators for testing. Even though the threshold for average variance extracted (AVE) is 0.5 or higher [68], a combination of an AVE of 0.4 or higher, with a composite reliability (CR) of 0.6 or more [69], which makes the coefficients of this study appropriately reliable and acceptable. The internal consistencies of each factor captured by Cronbach’s α are over 0.81, making them accepted because the allowed value is 0.7 or higher [70]. To sum up, the four extracted factors meet the standards requirements and are sufficiently reliable. Accordingly, these data will be further analyzed by SEM to analyze the correlation between the tested variables.

### 4.2. Results of SEM

Table 2 presents the estimated results obtained using the SEM. The results reveal that consumer preference for product modularization (*p* < 0.01) and inter-consumer connectivity (*p* < 0.001) have a positive significant impact on factors that make consumers realize the value of smart home products, while consumers who could perceive the value of platform services have no significant impact on the value of smart homes. The estimated results show that the interaction effect (0.564) is greater than the modularization effect (0.277). In order to test the reliability of SEM results, we referred to previous studies about smart homes that used SEM method [15,19]. Therefore, we selected three indicators to test the results of SEM. The applicability of these models was also evaluated (Table 3). Three general indicators were used to measure the suitability of the model: the root-mean square error of approximation (RMSEA) is 0.04 which is less than 0.06 [71]; the incremental fit index (IFI) is 0.96 which is more than 0.90 [72]; and finally, the comparative fit index (CFI) is 0.96, which is more than 0.90 [72]. The tests revealed that the indicator meets the requirements and justifies the use of the model.

The previous studies proposed that platform service and modularity can bring convenience to consumers. For example, the management of the smart home by platform service enhances the convenience of consumers; modular products can satisfy consumers’ various choices; additionally, inter-consumer connectivity can enhance consumers’ immersion [46,49,58]. In the results of this study, we can find that modularity and inter-consumer connectivity have a significance with consumers’ value perception, which is consistent with previous studies. Contrary to the assumptions of previous studies, it was suggested that the value recognition of centralized platform services is not related to the value recognition of smart home products. The proposals of previous research on the centralized management platform services are only from the developer’s point of view and are only assumptions; since consumer surveys were conducted, the discovery of this research set a precedent in this sense [16,27]. Therefore, we considered that this contradiction could occur.

**Figure 3:**
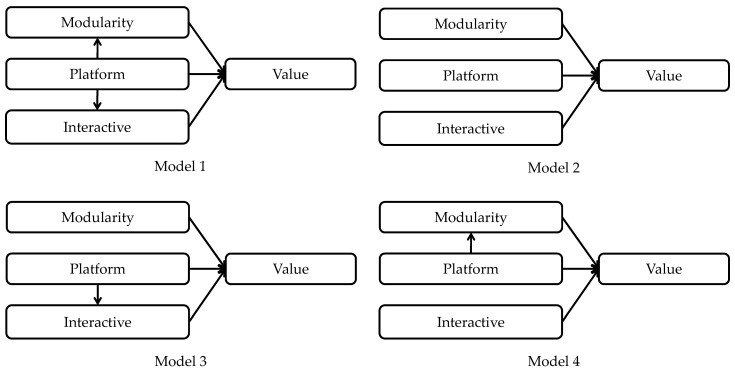
Alternative models.

Based on the results shown in Table 2, the platform service had no direct impact on the consumers’ value perception on smart home products. However, the possibility that it has an indirect impact through other variables was considered. Therefore, additional verification concerning it was performed. In order to explore this, the study evaluated four models (as shown in Figure 3) and confirmed which was the most appropriate model by referring to a research method from a previous study [73]. In Figure 3, the absence of an arrow from the platform service to the other two elements means that its direct route is limited to 0. As a result, the model fitting indices for each model are shown in Table 4. According to the comparison of various indicators, the indicators such as “Chisq./df”, “RMESA”, “SRMR”, “CFI”, “Aic”, and “Ecvi” of Model 1 are the smallest. Compared with Model 1, after removing the direct path, the fit of the other three models was significantly reduced. According to the results given, it can be understood that Model 1 was the most suitable. 

**Figure 4:**
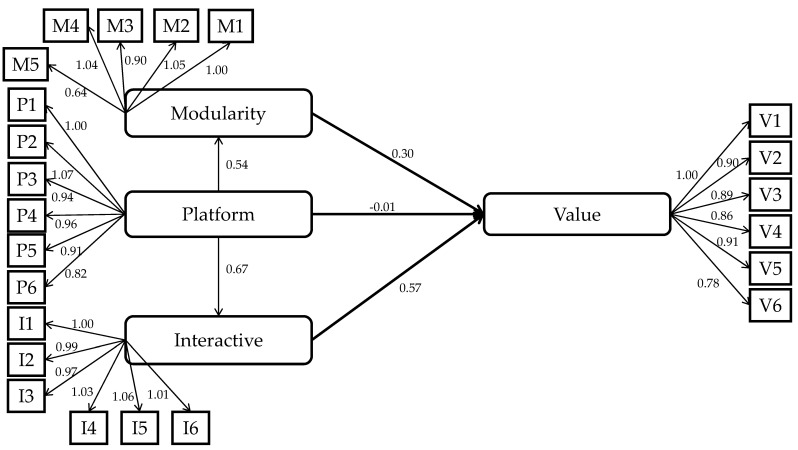
The structural equation model regarding the indirect impact of the platform service on the value perception of smart home products.

Table 5 shows the analysis results of Model 1. The results show that through modularization and interaction, consumers who value the platform service can perceive the value of smart home products (both *p* < 0.001). Figure 4 shows the structural relationship between the variables in the case of Model 1. Hence, the platform service has an indirect impact on consumers via modularity and inter-consumer connectivity. For consumers who can perceive the values of modularity and inter-consumer connectivity, the platform service can better make them feel the value of both elements. In such a way, the platform service can indirectly make these consumers feel more the value of smart home products. 

## 5. Discussion

In this study, the data of consumers related to smart home products were gathered using an online questionnaire survey. The design of the questionnaire considers factors related to the platform ecosystem of smart home products. Factor analysis and SEM were conducted to confirm whether consumers interested in the value of the relevant elements of the platform ecosystem could recognize the value of smart home platform products. The results showed that consumers who can perceive the value of product modularity and inter-consumer connectivity can also better realize the value of smart home products. Nevertheless, the results also confirm that consumers who can perceive the value of platform services have no significant influence on the perception of the value of smart home products. The results can be interpreted as specified below.

First, as revealed in previous studies, consumers who perceive the value of modularization can perceive the value of their products [7,50]. In other words, consumers can perceive the value of their products by distinguishing between several products based on their complements. The results of this study suggest that the benefits of modularization, which have been presented in previous studies, may also appear in the construction of a smart home platform ecosystem. 

Second, previous studies claimed that communication between consumers plays an important role in value perception [7,58]. Consumers can create value through direct network effects [55]. The communication between consumers is flexible which enables more easily meeting the needs of consumers [58]. By exchanging experiences, consumers can more flexibly determine how to use products that meet their needs. Therefore, similarly to the results of modularization, the benefits of consumer interaction, as presented in previous studies, may also appear in the construction of the smart home platform ecosystem. 

Third, previous studies suggested that consumers who perceive the value of platform services perceive the value of their products [30,47]. Some differences between the results of this study and previous studies concerning the platform service may be found since previous studies on platform services did not focus on the smart home industry. The results of this study indicate that the platform service variable had no significant direct impact on consumers. In the smart home industry, consumers use services provided by platform providers. However, the expression of such services is typically reflected in the smart home products—not in the platform itself. In other words, consumers in this industry might not visualize the linkage between the value from platforms and value from smart home products. On the other hand, this study also showed that consumers who can perceive the value of the platform service can indirectly feel the value of smart home products through modularity and inter-consumer connectivity. A previous study suggested that the platform service grants consumers more flexibility when using modular products, as well as to improve stability and other features which will give consumers a better experience [74]. In addition to this, the results of this study suggested that the functions related to platform service could be effective in the smart home markets, only if the smart home products have functions related to modularity or inter-consumer connectivity. In this case, the most direct source of consumer experience becomes modularized features and interactions with other consumers installed in the smart home products—not in the platform service. Therefore, modularity and inter-consumer connectivity provide an intermediation to experience platform service for consumers who value the platform service. 

### 5.1. Theoretical Implications

#### 5.1.1. Contribution to Platform Ecosystem Research

This research complements previous studies on smart home platforms. In this study, platform ecosystem development was examined from the perspective of consumers. Previous studies mainly explored conditions to meet the needs of consumers [8,9]. This study developed a theory of consumer value perception from related theories of the smart home industry and platform ecosystem [57,58]. The results of this study indicate that two platform-related factors, namely modularity and inter-consumer connectivity, can affect consumers’ sense of value for smart home products. Therefore, the indirect network mechanism between complements and consumers [7] as well as the direct network mechanism among the consumers themselves [7] can comply with the smart home platform ecosystem. The results also showed the that platform service does not directly affect consumers’ value perception of the product. In contrast, consumers who can perceive the value of platform service can indirectly feel the value of smart home products through modularity and inter-consumer connectivity. These findings could provide inspiration and directions for future research on platform ecosystems. 

#### 5.1.2. Contribution to Smart Home Research

This study contributed to the integration of Smart Home 2.0 and platform ecosystem theory related to IoT services. Compared with the Smart Home 1.0 era, the Smart Home 2.0 era is premised on strengthening the connection among products [4]. Due to these characteristics, Smart Home 2.0 needs to consider the perspective of the platform ecosystem. However, in previous studies, the impact on Smart Home 2.0 was not studied from the perspective of platform ecosystems. This study used factor analysis and SEM to analyze the three elements of the platform ecosystem associated with consumers’ perception of value for smart home products. The results presented in this paper revealed that in the Smart Home 2.0 era, consumers who can perceive product modularity and inter-consumer connectivity can more clearly perceive the value of smart home products. In addition, this study indicates that consumers interested in the platform service can be indirectly affected regarding the value perception of products through modularity and inter-consumer connectivity. Therefore, the statistical analysis enabled studying the development conditions of the Smart Home 2.0 platform ecosystem. Through the study of Smart Home 2.0, this study reflects the continuous progress of the smart home industry and contributes to the development of theory on smart homes.

### 5.2. Managerial Contributions

The results presented in this study show that consumers who can perceive the modularity of products and the value of inter-consumer connectivity can better perceive the value of smart home products. Furthermore, it was revealed that a platform service has an indirect impact on the value perception of smart home products. The paper provides new ideas for the management and development of smart home platforms concerning IoT services. As the main driving factor of satisfaction and intention, product value can help enterprises better allocate resources and gain a competitive advantage by studying consumers’ value perception [75]. 

#### 5.2.1. Implications on Platform Service

This study showed that although platform service has no direct impact on consumers’ perception of value on smart home products, it can indirectly influence perception through modularity and inter-consumer connectivity. This result implies that smart home platform owners should strengthen the service support for modularity and inter-consumer connectivity. Enhancing support for these two aspects will not only make consumers interested in platform service feel the value of platform service from smart home products, but also improve the level of modularity and inter-consumer connectivity to create more platform value. For example, in order to make consumers interested in platform service feel the convenience brought by platform service, enterprises can advance the stability of modular products by increasing platform services [75]. 

#### 5.2.2. Implications on Modularity

In the Smart Home 2.0 era, one of the goals is to create a more comfortable home environment [4]. Smart home platforms need to increase opportunities for complementary product development and strengthen the development of smart home modularity. This research also revealed that consumers who can perceive the modularity of products can better perceive the value of smart home products. Thus, the modularity of products is effective for these consumers to perceive the value of smart home products. As far as smart home platforms are concerned, smart home products are becoming more dispersed and miniaturized. A platform not only involves the technology or service of a company, but also a complementary ecosystem usually produced by various businesses [76]. Modularity can allow more industries to participate, thus providing more unique services. The products and functions of different companies differ from each other. The companies interact and learn from each other to ensure the continuous operation of smart home platforms. Moreover, innovation ability, interconnectivity, complementarity, efficiency and network effectiveness are the five capabilities of platform owners which lead to the platform [77]. Smart home products are modularized to ensure interconnectivity and complementarity between products. Modular products can complete a set of more complex functions through more free combinations, thereby improving the use efficiency for consumers in a way that consumers who are interested in modularity can feel the value of the product. In the case of product modularity, more enterprises can join the platform, expand and develop the platform with a complete system.

#### 5.2.3. Implications on Inter-Consumer Connectivity

Cooperation among consumers can increase the value of the platform [55]. Therefore, promoting inter-consumer connectivity will increase the value of the platform and allow more consumers to participate in it. This study shows that consumers who can perceive the value of inter-consumer connectivity can better perceive the value of smart home products. This can also encourage consumers to more actively participate in the improvement of products and services while providing more suggestions for products and services of the platform [54]. By doing so, one can assist the consumer in enjoying better services. As these consumers have higher expectations for smart home products, enhancing the inter-consumer connectivity is a viable option for platform companies and product developers, and thus leading to an increase in the degree of cooperation between consumers will lead them to rely more on products and become more loyal to the platform in use.

### 5.3. Limitations and Future Research

This study focused on the ability of Japanese consumers to perceive the value of smart home products. It also focused on the way smart home platforms can adapt to the Smart Home 2.0 era in a way that platform owners can maintain and expand their platforms. Nevertheless, this study has some limitations. 

First, while examining the elements related to the platform, issues that consumers pay attention to could not be included. There are still some factors that affect the ease of use and versatility [19]. For instance, in the smart home business, the Wi-Fi function and the functionality of connecting with external products have an obvious influence on the versatility and usability of products [17]. Therefore, it is necessary to conduct more detailed research on the usability and universality of the factors affecting these consumers. Factors related to customization, the use of personal information, and factors related to the platform may interact with each other to draw a more comprehensive conclusion. Thus, future research can study the improvement of products and services and contribute to service business-related theories.

Second, this study was a comprehensive study of platform factors. Platform providers, complements, and consumers are not the main targets. Future studies should more extensively examine these three aspects in order to understand which factor will affect the platform ecosystem. For instance, the synergy between complements should be studied to gain a more comprehensive understanding of the interaction between complements and their content. Thus, future research can supplement the interactions among the elements of the platform ecosystem.

Finally, given its focus on Japanese consumers, this survey may not represent consumers in other regions. The development process of smart homes in each region is different; therefore, more comparative research is necessary. By examining other markets of different countries and comparing them with the Japanese market, the applicability of this theory could be globally tested, thus making it universal and more credible.

## 6. Conclusions

This study aimed to discuss the impact of platform system elements on the consumer perception of value for smart home products. The study was based on 595 samples for factor analysis and SEM analysis in order to test whether consumers who can perceive the value of the platform ecosystem elements can perceive the value of smart home products. The results show that consumers who can perceive modularity and inter-consumer connectivity can perceive more value in smart home products. Moreover, for consumers who can perceive the value of a platform service, they can indirectly perceive more value in smart home products through modularity and inter-consumer connectivity. This study showed that modularity and interaction have a direct impact on consumer’s value perception. Platform services have an indirect impact on the consumer perception of value through the other two platform ecosystem elements. Therefore, this study indicated that improving these platform ecosystem elements is important for the development of smart home products. Finally, contributions of this study for the product development in Smart Home 2.0 is shown in Table 6.

## Figures and Tables

**Table 1:** Factor analysis results and factor reliabilities.

Question Item	Value	Platform	Modularity	Interactive
*For the dependent variable*				
V1	0.72			
V2	0.68			
V3	0.62			
V4	0.65			
V5	0.68			
V6	0.60			
*For the explanatory variables*				
P1		0.76		
P2		0.80		
P3		0.71		
P4		0.63		
P5		0.73		
P6		0.63		
M1			0.75	
M2			0.74	
M3			0.66	
M4			0.77	
M5			0.47	
I1				0.69
I2				0.71
I3				0.68
I4				0.73
I5				0.71
I6				0.71
Average variance extracted	0.43	0.51	0.47	0.49
Composite reliability	0.82	0.86	0.81	0.85
Cronbach’s α	0.82	0.86	0.81	0.85

Note: Here, “V” stands for the dependent variable “Value” in the questionnaire. “P” stands for the items in the questionnaire on the explanatory variable “Platform”. “M” stands for the items in the questionnaire describing the “Modularity” explanatory variable. “I” stands for the questionnaire items describing the “Interaction” explanatory variable. Average variance extracted is a measure of the amount of variance captured by the structure [68]. Composite reliability is a measure of internal consistency in scale items [69]. Cronbach’s α is considered as an indicator of internal consistency [70].

**Table 2:** Estimation results.

	Estimate	Std. Error	*p* Value
Platform~Value	0.020	0.076	0.793
Modularity~Value	0.277	0.083	0.001
Interactive~Value	0.564	0.070	0.000

**Table 3:** Model-fit indices.

	RMESA	IFI	CFI
Level	0.04	0.96	0.96
Acceptance level	<0.06	>0.90	>0.90

Note: RMESA = root-mean square error of approximation; IFI = incremental fit index; CFI = comparative fit index.

**Table 4:** Fit indices among competing models.

	Chisq.	Df	Chisq./df	RMESA	SRMR	CFI	Aic	Ecvi
Model 1	465.467	225.000	2.069	0.044	0.052	0.950	33194.650	1.049
Model 2	793.020	227.000	3.493	0.068	0.182	0.883	33518.214	1.647
Model 3	680.589	226.000	3.001	0.061	0.148	0.906	33407.782	1.443
Model 4	589.015	226.000	2.606	0.054	0.140	0.925	33316.209	1.274

Note: Chisq. = chi-square value; df = degree of freedom; RMSEA = root-mean square error of approximation; SRMR = standardized root-mean square residual; CFI = comparative fit index; AIC = Akaike in formation criterion; ECVI = expected cross-validation index.

**Table 5:** Estimation results for Model 1.

	Estimate	Std. Error	*p* Value
Platform~Value	−0.006	0.084	0.943
Modularity~Value	0.304	0.083	0.000
Interactive~Value	0.574	0.070	0.000
Platform~Modularity	0.639	0.054	0.000
Platform~Interactive	0.545	0.057	0.000

**Table 6:** Summary of contributions.

Results	Contributions for the Product Development in Smart Home 2.0
Consumers who perceive the value of “platform service” can perceive more value of smart home products through modularity and inter-consumer connectivity.	Consumers who perceive the “platform service” have higher expectations for smart home products. However, this relationship is indirectly realized through expectations for “modularity” and “inter-consumer connectivity”.
Consumers who perceive the value of “modularity” can perceive more value of smart home products.	Consumers who perceive the “modularity” of products have higher expectations for smart home products. This could suggest legitimacy to develop more modularized smart home product in the Smart Home 2.0 era.
Consumers who perceive the value of “inter-consumer connectivity” can perceive more value of smart home products.	As the consumers who perceive “inter-consumer connectivity” have higher expectations for smart home products, enhancing the inter-consumer connectivity could be a viable option for platform companies and product developers of the future smart home market.

## Data Availability

The data presented in this study are available on request from the corresponding author.

## Appendix A

Table A1 lists the questionnaire items. A five-step evaluation of one-to-one comparison was conducted based on the three explanatory variables presented in this study. For the dependent variable “Needs”, option A was set to “the respondents appreciate the value related to smart home products”. Option B stands for “the respondents think traditional products are sufficient”. For the explanatory variable “Platform”, option A means “the respondents appreciate platform services provided by platform companies”. Option B stands for “the respondents think in terms of individual use of smart home products”. For the explanatory variable “Modularity”, option A means “the respondents appreciate modularization of smart home products”. Option B stands for “the respondents appreciate the unified management of smart home products”. For the explanatory variable “Interactive”, option A means “the respondents feel valued in their communication with others”. Option B stands for “the respondents think it is enough to use it by themselves”.

**Table A1:** Questionnaire items.

Code Name	Option A	Option B
V1	Set the temperature with smart refrigerators, and check the shelf life of food with smart phones.	Satisfied with the use of ordinary refrigerator.
V2	By using the intelligent washing machine, the dosage of powder detergent and liquid detergent can be automatically controlled.	The function of the washing machine has been sufficient so far.
V3	Smart scales can manage the health data of separated families and can take care of their family health without meeting each other.	It is sufficient to use an ordinary weight scale for weight measurement.
V4	Intelligent electric toothbrushes can be used to display the status of brushing the teeth and diagnose whether it is good or bad.	Satisfied with the toothbrush used after brushing.
V5	With a smart rice cooker, it is possible to set the timing of rice cooking through smartphone on the go.	So far, the function of rice cooker is very good.
V6	Intelligent ceiling lamps can automatically control dimming by connecting to other devices in order to create an appropriate atmosphere in the room.	Controlling the lamp with one’s own hands is suffice.
P1	Centralized management of smart home products on the platform enables usage of a variety of products and their usage simultaneously when needed.	Smart home products are purchased with a purpose, so there is no feeling regarding the need for mutual cooperation.
P2	Platform allows all smart home products to be connected through the platform, which saves the trouble of establishing connections.	Integration of smart home products should be handled separately as required.
P3	Smart home products connected to a platform can be controlled using the unified application program (interface) of the platform	Operating each smart home product with a separate application is not particularly troublesome.
P4	Uploading personal information required by the platform eliminates the need for individual uploads to connect to smart home products.	If necessary, personal information should be uploaded independently.
P5	Consult the service desk of the platform company for help regarding smart home products that can be connected to.	For product support, one should consult the support department in the product’s company.
P6	Products that can be connected to the platform can be purchased through the website.	When purchasing a smart home product, product collection and buying information should be on the websites of each brand.
M1	Expanding the ability of intelligent service should be through expansion.	The service function of the product may remain as it was at the time of purchase.
M2	The ability of expansion and cooperation with other products.	A function that is complete within a single product should be provided.
M3	Each product has few functions and can be used alone, but with the option to buy a large number of products to meet the needs and work together.	One general-purpose product that can meet general needs is suffice.
M4	Expand user’s capabilities by connecting to other devices.	Features are well contained in a single product, but cannot be connected to the outside world.
M5	Each product has specific functions, and there is no overlap between the functions of the products.	Versatility cannot help but overlap features between products.
I1	If an acquaintance is using intelligent health services, the results of their use can be seen and will motivate the users to improve their health.	It is enough to use the health service function to check my own health.
I2	An option for a video conference or bulletin board function on the smart TV, can improve communication with any acquaintance of the user.	Smart TV should be able to surf the Internet, such as YouTube.
I3	The option to easily talk with online with acquaintances using the intelligent artificial intelligence systems is very convenient.	The intelligent artificial intelligence systems are sufficient to have artificial intelligence assistance capabilities.
I4	A mechanism to cook and exchange information with an expert in a smart kitchen would be nice.	Just using the ready-made menu is enough for cooking.
I5	The usage of intelligent security with family members and acquaintances enable to understand the situation at home.	Intelligent security is enough to prevent crime.
I6	The possibility to listen to the same music with family members and friends using smart speakers, brings more enjoyment.	It is sufficed if smart speakers could be used to enjoy music alone.

Note: Here, “V” stands for the dependent variable “Value” in the questionnaire. “P” stands for the questionnaire items for the explanatory variable “Platform”. “M” stands for the questionnaire items describing the “Modularity” explanatory variable. “I” stands for the questionnaire items describing the “Interaction” explanatory variable.
